# Assessment of early lymphopenia after cardiac arrest

**DOI:** 10.1186/2197-425X-3-S1-A196

**Published:** 2015-10-01

**Authors:** P Villois, V Fontana, C Righy Shinotsuka, K Donadello, JL Vincent, J Creteur, FS Taccone

**Affiliations:** Erasme University Hospital, Bruxelles, Belgium

## Introduction

A significant decrease in circulating lymphocytes can occur after septic shock and severe trauma and is associated with a poor outcome. After cardiac arrest (CA), the return of spontaneous circulation is associated with a significant inflammatory response that can also mimic the same alterations, but lymphocyte count has not been studied in this setting.

## Objectives

To investigate whether circulating lymphocyte count is associated with patients' outcome after CA.

## Methods

Retrospective analysis of an institutional database including all adult comatose patients admitted to the Intensive Care Unit (ICU) after CA from January 2007 to December 2014. Inclusion criteria were as follows: age ≥18, non-traumatic arrest and survival ≥ 24 hours after admission. Demographics, CA-related findings and outcome information were included in the database. We collected data on daily routine blood analyses and, in particular, total white blood cells (WBC) count, neutrophils and lymphocytes. Lymphopenia was defined as a lymphocyte count < 1000/mm^3^. We recorded ICU mortality and long-term neurological outcome; a CPC score of 3-5 at 3-months was used to define poor neurological outcome (PNO).

## Results

Of the 404 eligible patients, 27 were excluded because of lack of blood analyses and 377 were eventually included. Median age was 62 [52-75] years and 263 (70%) were male. Median time to ROSC was 15 [8-25] minutes and 232 (62%) had a non-shockable initial rhythm. ICU mortality was 58% (n = 217) and 3-month PNO was observed in 246 (65%) patients. Median WBC count on the day of admission was 12200 [9000-16700]/mm^3^, including a lymphocyte count of 1208 [700-2350]/mm^3^. Of these, 199 (53 %) had lymphopenia, which was moderate in 151 and severe ( < 500/mm^3^) in 48. Thirty-nine (25%) patients had recovered a normal lymphocytes count within the first 2 days in the ICU. Lymphocyte count was lower in patients under immunosuppressive therapy (n = 77) than in the others (p = 0.004), and in patients with in-hospital CA (n = 164) when compared to out-of-hospital CA (p < 0.001), as well as in patients with non-shockable rhythms when compared to shockable ones (p < 0.001). Moreover, patients developing an infection (n = 215) had also lower lymphocytes on admission than the others (p = 0.01). Finally, ICU non-survivors had lower lymphocytes than survivors (1100 [613-2317] vs. 1316 [891-2395]/mm^3^; p = 0.05) as well as patients with PNO when compared to those with favourable outcome (1100 [600-2013] vs. 1350 [919-2614]/mm^3^; p = 0.003).

## Conclusions

After CA, lymphocyte count is lower in patients with non-shockable rhythms, in-hospital CA and previous immunosuppressive therapy than in the others. A lower lymphocytes count was also found in non-survivors and in patients with poor neurological outcome.

